# Correlation factors for distal syndesmosis ossification following internal fixation of ankle fracture

**DOI:** 10.1038/s41598-018-30672-7

**Published:** 2018-08-23

**Authors:** Lu Bai, Wen Zhou, Wentao Zhang, Jianxin Liu, Honglei Zhang

**Affiliations:** 1 Department of Sports Medicine Shenzhen Hospital of Peking University, Shenzhen, 518036 Guangdong China; 2 Department of Radiology Shenzhen Hospital of Peking Uiversity, Shenzhen, 518036 Guangdong China; 3 Department of Rehabilitation Shenzhen Hospital of Peking University, Shenzhen, 518036 Guangdong China

## Abstract

This clinical retrospective study explored factors associated with distal tibiofibular syndesmosis ossification (TFSO) after ankle fracture fixation. Between August 2012 and January 2015, 172 patients with ankle fractures (121 men) with an average age of 46.6 years (range, 22–71 years) were treated surgically with an average follow-up period of 26 months (range, 16–34 months). According to the Danis-Weber AO classification rubric, 54 fractures were type A, 78 were type B, and 40 were type C. According to the Lauge-Hansen classification, there were 17 supination-adduction (SA) fractures, 98 supination-external rotation (SE) fractures, 31 pronation-external rotation (PE) fractures, and 26 pronation-abduction (PA) fractures. The average injury to operation interval was 4.3 days (6 hours-7 days). Multiple factor analysis was conducted to examine risk factors for TFSO. It was observed in 36 (20.9%) cases (11 complete ossification cases; 25 partial ossification cases). Multivariate logistic regression revealed the following independent risk factors for TFSO were: AO classification, distal tibiofibular syndesmosis separation, and fibular fracture morphology. In conclusion, AO type C fracture, syndesmosis separation, and high fibular fracture were associated with distal TFSO following ankle fracture fixation.

## Introduction

Ankle fracture is a common clinical finding, with recent epidemiological data showing occurrence rates of 120–150/100,000^1–4^. The occurrence rate of ankle fracture is increasing, particularly among older patients due to aging associated increases in fragility fractures^5,6^. Anatomical reduction, rigid fixation, and early functional rehabilitation have become classical principles for the treatment of ankle fracture, and surgical fixation based on these principles has achieved optimal outcomes^7–10^. Tibiofibular syndesmosis ossification (TFSO) after surgical treatment of ankle fracture occurs in about 3–7% of cases^11^, with a rate exceeding 10% in a large case series^12^. However, reports on tibiofibular syndesmosis synostosis are currently inadequate.

Early research showed that calcification of the anterior inferior tibiofibular ligament after distal tibiofibular syndesmosis sprain was the main cause of ossification^13^. Some recent case reports have suggested that tibiofibular syndesmosis separation and screw fixation are independent risk factors for postoperative synostosis^12,14,15^. However, no definitive conclusion has been reached about the main causes of such ossification or whether it is related primarily to the severity of the primary injury or the surgical treatment (e.g., syndesmosis screw use). We conducted a radiological study and retrospective analysis of surgically treated ankle fractures in the past 3 years to examine relevant risk factors for the complication of synostosis. We hypothesized that TFSO would be related to the morphology of the fibular fracture and to screw fixation.

## Results and Discussion

The sample was comprised of 172 patients (121 men, 51 women) who were treated for ankle fracture, with a mean age of 46.6 years (range, 22–71 years); 74 (43%) patients were >50 years old. The cohort included 64 underweight patients (37.2%), 82 normal-weight patients (47.7%), and 26 overweight patients (15.1%). With respect to comorbidities, 35 (20.3%) patients had systemic diseases, including 19 (11.0%) with hypertension, 13 (7.6%) with type 2 diabetes, 2 (1.2%) with gout, and 1 (0.6%) with chronic rheumatoid arthritis.

According to the AO (Danis-Weber) classification system, 54/172 fractures (31.0%) were type A, 78 (45.3%) were type B, and 40 (23.3%) were type C. According to the Lauge-Hansen classification system, the sample included 17 (9.9%) supination-adduction (SA) fractures, 98 (57.0%) supination-external rotation (SE) fractures, 31 (18.0%) pronation-external rotation (PE) fractures, and 26 (15.1%) pronation-abduction (PA) fractures. The deltoid ligament was intact in 158 patients (91.9%) and ruptured in 14 patients (8.1%). Wound infection (treated with continuous dressing until the wound healed) and superficial nerve injury were present in 8 patients (4.7%) and 9 patients (5.2%), respectively. There were 40 cases (43.3%) of distal tibiofibular syndesmosis separation, of which 39 (97.5%) were diagnosed as ankle-joint dislocation, with an average post-injury operation period of 4.3 days (range, 6 hours-7 days).

All patients were treated with open reduction and internal fixation; the posterolateral approach to the lateral malleolus was used in 20 cases, and the lateral approach was used in the remaining cases. Among 48 posterior malleolar fractures, 25 were treated with cannulated screw fixation. No fixation was performed after restoration in the remaining 23 cases, as the fracture involved <25% of the articular surface. All cases of deltoid ligament rupture were sutured and fixed with an anchor. Distal tibiofibular syndesmosis injuries were fixed with single screws; three and four cortical screws were used in 12 and 28 cases, respectively. Fixation splints or braces were used for 3 weeks in 56 patients for fixation.

The average interval between surgery and final radiography was 14.7 months (range, 7–33 months). Final radiographs showed TFSO in 36 (20.9%) patients, including complete TFSO in 11 cases (30.6%) and partial TFSO in 25 cases (69.4%). Of the 36 TFSO cases, ossification occurred through the fracture line in 14 cases (38.9%) and through the screw in 22 cases (61.1%).

Univariate analysis showed that AO type B and C fractures were more common than type A fracture. Synostosis was more common in patients with, than in those without, tibiofibular syndesmosis injury (Table 1). The rate of ossification differed significantly between cases involving ankle dislocation and those that did not (Table 1). Multiple logistic regression analysis identified AO classification, tibiofibular syndesmosis separation, and fibular fracture morphology as independent risk factors for TFSO(Table 2).

**Table 1:** Univariate analysis of variables potentially associated with inferior TFSO.

Variable		Ossification			*X* ^2^	*P*
		None	Partial	Complete		
Sex	Male	96	17	8	0.100	0.951
	Female	40	8	3		
Age group (years)	<50	75	13	10	5.605	0.061
	>50	61	12	1		
Body mass index	<18.5	49	11	4	5.516	0.238
	18.5–25	69	10	3		
	>25	18	4	4		
AO fracture type	A	52	1	1	21.803	<**0**.**001**
	B	61	13	4		
	C	23	11	6		
Lauge-Hansen fracture type	SE	78	15	5	1.106	0.981
	SA	14	2	1		
	PE	24	4	3		
	PA	20	4	2		
Deltoid ligament	Intact	126	22	10	0.624	0.732
	Ruptured	10	3	1		
Posterior malleolus	Intact	98	18	8	0.002	0.999
	Fractured	38	7	3		
Posterior fixation	No	117	20	10	0.898	0.638
	Yes	19	5	1		
Syndesmosis	Intact	113	14	5	15.129	**0**.**001**
	Separated	23	11	6		
Syndesmotic screw	None	113	14	5	15.129	**0**.**001**
	One	23	11	6		
Syndesmotic cortical screw	None	113	14	5	21.443	**0**.**001**
	Three	4	5	3		
	Four	19	6	3		
Reduction of syndesmosis	Anatomic	130	22	9	4.758	0.093
	Malreduction	6	3	2		
Ankle dislocation	No	110	18	5	7.758	**0**.**023**
	Yes	26	7	6		
Cast immobilization	No	96	15	5	3.666	0.160
	Yes	40	10	6		
Comorbidities	No	106	20	11	3.057	0.217
	Yes	30	5	0		
Fibular approach	Lateral	121	21	10	0.581	0.748
	Posterolateral	15	4	1		
Wound infection	No	131	24	9	4.856	0.088
	Yes	5	1	2		
Superficial nerve injury	No	118	22	8	1.763	0.414
	Yes	18	3	3		
Fibular fracture morphology	Trans-Syndes	98	19	6	1.821	0.402
	High position	38	6	5		

SE, supination–external rotation; SA, supination-adduction; PE, pronation–external rotation; PA, pronation-abduction.

**Table 2:** Logistic regression analysis of potential risk factors for TFSO.

Factor	*β*	SE	Wald	*P*	OR	95% CI	
						Lower	Upper
Sex	−0.03	0.5	0	0.95	0.97	0.37	2.57
Age group	−0.03	0.01	4.85	0.03	0.97	0.94	1
Body mass index	−0.27	0.53	0.26	0.61	0.76	0.27	2.17
Syndesmosis injury	−0.77	0.26	9.47	**0**.**003**	3.83	3.17	9.96
Lauge-Hansen type	0.67	0.71	0.88	0.35	1.95	0.93	3.11
Fibular fracture morphology	−5.15	1.98	6.76	**0**.**01**	2.15	1.78	4.28
AO classification	1.42	0.32	19.40	**0**.**0001**	4.14	2.20	7.80
Deltoid ligament	0.1	0.81	0.01	0.90	1.1	0.23	5.38
Posterior malleolus	0.22	0.66	0.11	0.74	1.25	0.34	4.55
Ankle dislocation	1.44	0.78	3.22	0.16	2.72	0.67	11.04
Cast immobilization	0.1	0.66	0.02	0.88	1.11	0.3	4.06
Comorbidities	−0.69	0.69	0.99	0.32	0.5	0.13	1.94
Fibular approach	0.47	0.82	0.32	0.57	1.59	0.32	7.96
Constant	−1.08	0.92	1.37	0.24	0.34	—	—

SE, standard error; OR, odds ratio; CI, confidence interval;

The distal tibiofibular syndesmosis is a stable structure composed of the distal tibia and the incisura and convex side of the distal fibula, connected by anterior and posterior ligaments and an interosseous membrane. This joint contains no cartilage. The inferior tibiofibular syndesmosis is a micro-motion joint with a 2–4° range of motion that allows the ankle bones with different anteroposterior diameters to match the joint contact area during ankle motion^16,17^.

In ankle fractures, rotational trauma is transmitted to the distal tibiofibular syndesmosis through the lateral or medial malleolus, causing injury and separation. Treatment modalities include anatomical reduction and strong fixation of the ankle fracture and inferior tibiofibular syndesmosis. Although surgical treatment of ankle fractures achieves good results, inferior TFSO develops postoperatively in some patients. Although some scholars believe that such ossification may affect ankle function^18,19^, Hinds *et al*.^12^ found no obvious effect on ankle function score in a study of 564 ankle fracture cases; they did, however, find that dorsal stretching, plantar flexion, and internal rotation were restricted to some extent. Because distal syndesmosis ossification is a relatively rare complication, its causes are not well documented. McMaster and Scranton^20^ hypothesized that it was related to tearing of the interosseous membrane, and Scranton *et al*.^21^ suggested that an inferior tibiofibular osseous connection is formed by the ossification of a hematoma after separation of interosseous membrane from the distal tibiofibular joint. In a retrospective analysis of ankle fractures with inferior tibiofibular injury, Kaye^22^ found an inferior tibiofibular osseous connection in 4/30 cases and ossification within the interosseous membrane in 3/40 cases. All cases with TFSO were treated with syndesmosis screws. Hinds *et al*.^12^ argued that ossification in the syndesmosis is irrelevant when the inferior tibiofibular syndesmosis is treated with screws, although the ossification area is close to the level of the fibular fracture, not the inferior tibiofibular syndesmosis. However, whether synostosis is related to degree of injury or treatment factors has not been determined.

As an uncommon complication of internal fixation of ankle fracture, syndesmosis ossification has not been described in reports on large series. In the present study, TFSO developed in the area of the fibular fracture line in 22 cases and around internally fixed screws in 14 cases. According to McKeon *et al*.’s^23^ anatomic study, the peroneal artery supplies blood to 67% (37/55) of inferior tibiofibular syndesmoses. The blood supply of the anterior ligament in the inferior tibiofibular syndesmosis comes mainly from the anterior perforating branch at the inferior end of the peroneal artery; this blood vessel has branches supplying the interosseous membrane when distributed at the anterior ligament in the syndesmosis, located about 3 cm from the adjacent end of the ankle perforation point. In addition, the anterior tibial artery supplies blood to 37% of the inferior tibiofibular syndesmosis. Thus, in low (within 3 cm of the proximal ankle mortise) ankle fractures, transverse or spiral fracture lines are likely to destroy peroneal artery perforators to the inferior tibiofibular syndesmosis, and local hematomas caused by perforator vessel hemorrhage may be a cause of TFSO.

In terms of morphology and mechanism, fracture adjacent to the tibiofibular syndesmosis may be a risk factor for ossification. In SE and PA (Lauge-Hansen classification), fracture lines pass through the syndesmotic area in oblique or transverse directions. In addition to the adjacency of a fracture to the syndesmosis being a factor in local vascular injury, hematoma, and subsequent heterotopic ossification, surgical procedures (e.g., drilling) in this area can also produce bone debris that leads to ossification. Surgical drilling can also damage nutrient-supplying blood vessels around the inferior tibiofibular syndesmosis, leading to hematoma formation.

In our sample, TFSO developed mostly at the fracture end in PE fractures, which are characterized by crushing. Crush injury may stimulate ossification due to the abundance of bone debris or growth factors contained in the local hematoma. Ossification resulting from hematoma may be a critical mechanism distinct from hectopic ossification in that a local hematoma does not create new bone morphogenetic protein factors that induce bone formation.

Whereas prior work has focused on ossification biomechanics or fixation type^24^, the present work was focused on factors related to syndesmosis ossification. Hinds *et al*.^12^ reported that male sex, ankle joint dislocation, and the use of screws in the inferior tibiofibular syndesmosis were the main reasons for ossification. In this study, patient sex had no significant effect, but syndesmosis injury was confirmed to be a risk factor for TFSO. In this case series, all syndesmosis separations were fixed with screws, and TFSO around the screws was common. However, whether this ossification was caused by the screws or injury of the inferior tibiofibular syndesmosis is unknown. No large trial has described synostosis with the use of endo-buttons. If screw use, rather than the injury, generates ossification, then stabilization of the distal tibiofibular syndesmosis with an endo-button, which involves cannulated drill use and therefore produces less bone debris, may reduce the occurrence rate of TFSO.

Anatomical research has shown that most inferior tibiofibular syndesmosis separations are backward dislocations, in which the distal end of the fibula moves backward and extorts relative to the tibia, tearing the inferior tibiofibular syndesmosis perforating branches at the peroneal artery. Rupture of the interosseous membrane further intensifies the formation of local hematoma, which creates conditions for heterotopic ossification. Because SA fracture does not involve damage to the inferior tibiofibular syndesmosis, ossification at this site is not typical. The single case of SA fracture ossification in the present study involved the screw pathway. This finding also suggests that distal tibiofibular syndesmosis injury is not the only factor leading to ossification.

This study was limited by the examination of a relatively small number of cases. Multicenter research is warranted. In addition, this study involved inferior tibiofibular syndesmoses fixed by screws, a simple treatment method. Because neither endo-buttons nor bio-absorbable screws were used, inferior tibiofibular syndesmosis injury was considered to be a risk factor, especially for heterotopic ossification. Whether screw use promotes local ossification remains unknown. In addition, this study did not include functional follow-up data from patients with and without TFSO.

In conclusion, data from our series suggest that syndesmosis separation is a likely cause of synostosis. AO type C fracture and high fibular fracture was also associated with risk of syndesmosis injury. We observed some ossification around screw pathways, which may represent another factor in synostosis risk.

## Materials and Methods

### Patients

The study protocol was approved by the ethical committee of Shenzhen Hospital Of Peking University. Written informed consent was obtained from all of the patients involved in the study. Before started, we did a power analysis to verify the number of cases. Under condition of alpha = 0.05, N = 20 to 200 by 10, OR = 3, R^2^ = 0.4, 2-tailed test. Power analysis Result: N = 170, Power = 0.80812.

Case inclusion criteria were: (1) ankle fracture treated surgically between August 2012 and January 2015 at the Peking University Affiliated Shenzhen Hospital, (2) availability of complete follow-up data, and (3) patient age ≥18 years. Exclusion criteria were: (1) occurrence of heterotopic ossification in another part of the body, (2) pathological fracture, and (3) brain injury concurrent with ankle fracture. Data from all cases were collected from the hospital’s database. According to the result of power analysis we take 172 cases into this research.

### Radiological assessment

**Figure 1:**
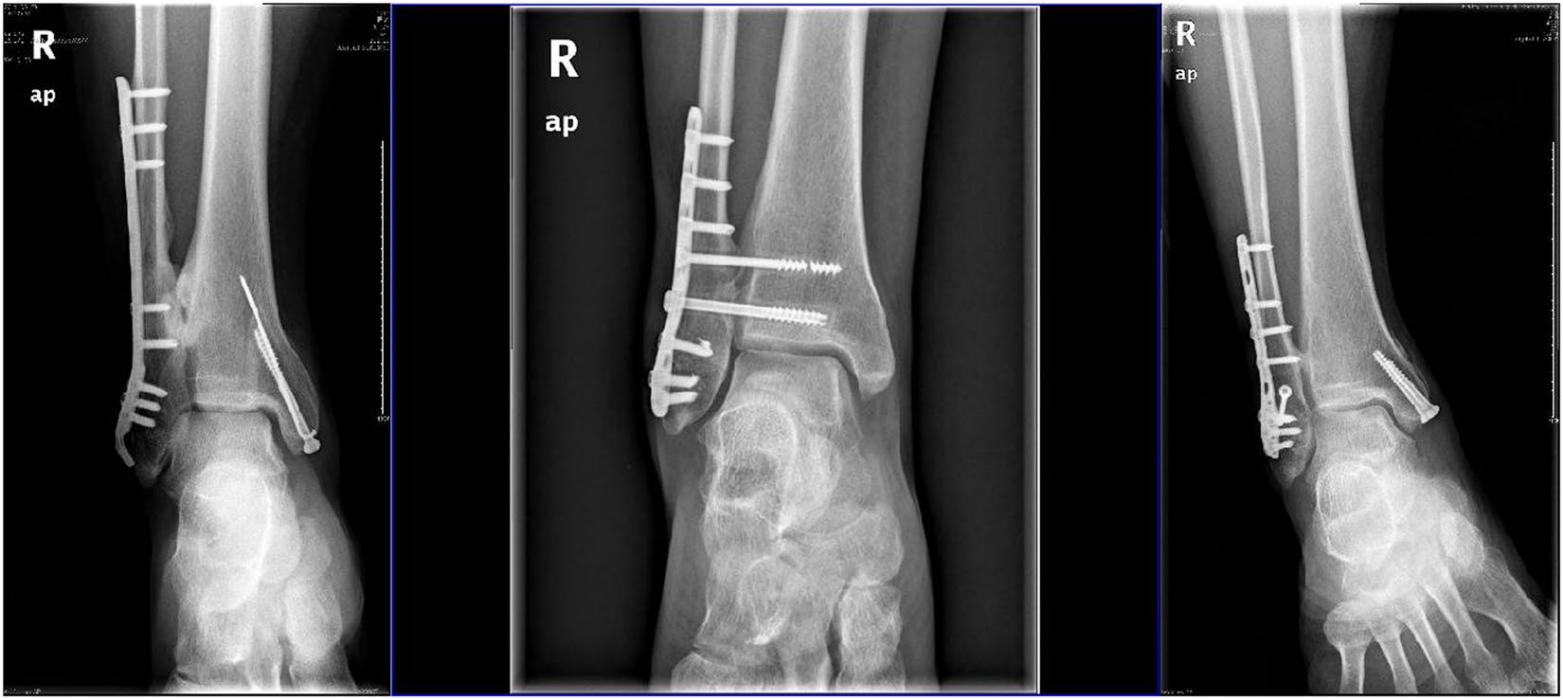
Ossification above the tibiofibular syndesmosis (high synostosis 1.1), synostosis around the tibiofibular syndesmosis (1.2), and ossification formation around a low distal fibular fracture and screws (1.3).

One experienced radiologist compared anteroposterior and lateral radiographs of each patient’s ankle taken immediately postoperatively with those obtained at the last follow-up visit (mean 26 months, range 16–34 months). The diagnostic criteria of Philips *et al*.^25^ were used to assess ossification. TFSO spanning the entire tibiofibular gap on radiographs was defined as complete, and TFSO that did not span the entire gap was defined as partial. To test the study hypothesis, we classified the location of synostosis as around the distal fibular fracture line or around the tibiofibular or fixed fibular screw (trans-syndesmosis). To examine whether TFSO was related to fibular fracture morphology, ossification sites were classified in relation to tibiofibular fractures as low (≤3 cm from the ankle line and tibiofibular joint) or high (>3 cm above the tibiofibular joint; Fig. 1).

### Statistical analysis

Statistical analysis was conducted in PASW 18.0 software (IBM, Armonk, NY, USA). Metric data are reported as means and standard deviations, and count data are reported as percentages. All statistical methods used have been recommended for this type of research^26,27^. First, analysis of possible risk factors for tibiofibular synostosis was conducted with univariate chi-squared tests. The analysis included the following demographic and clinical variables: age >50 years, sex, body mass index (underweight <18.5, normal 18.5–25, overweight >25), systemic disease, ankle joint dislocation, tibiofibular syndesmosis separation, AO and Lauge-Hansen classifications, deltoid ligament injury, and posterior malleolar fracture. The following treatment factors were also included: type of tibiofibular syndesmosis fixation (screws engaging three or four cortices), posterior malleolar fracture fixation, approach to the lateral malleolus, splint immobilization/fixation, and infection. Potential risk factors were then subjected to multiple logistic regression analysis. Statistical significance was set to *p* < 0.05.
